# 8 Less Is More: Reconsidering Hourly Urine Output Goals in Burn Resuscitation

**DOI:** 10.1093/jbcr/irac012.012

**Published:** 2022-03-23

**Authors:** Lauren B Nosanov, Kathryn E Harris, Kaitlyn M Libraro, Jeremiah P Lorico, Jamie M Heffernan, Philip H Chang, Abraham Houng, Jim Gallagher

**Affiliations:** New Year Presbyterian / Weill Cornell Medical Center, New York, New York; New Year-Presbyterian/Weill Cornell Medical Center, New York, New York; New York Presbyterian Hospital, New York, New York; New Year-Presbyterian/Weill Cornell Medical Center, New York, New York; NY Presbyterian Weill Cornell, New York, New York; New Year-Presbyterian/Weill Cornell Medical Center, New York, New York; Weill Cornell Medical College, New York, New York; none, phoenicia, New York

## Abstract

**Introduction:**

Significant morbidity and mortality is seen with high volume burn resuscitations. Surpassing the Ivy Index, defined as 250 milliliters/kilogram (ml/kg), has been correlated with increased incidence of complications such as abdominal compartment syndrome. Cognizance of factors contributing to over-resuscitation can help optimize fluid administration strategies to minimize associated morbidity.

**Methods:**

A single-center Quality Improvement review was performed of all adult (age ≧ 18 years) burn-injured patients presenting to a major metropolitan burn center with burns ≧ 20% total body surface area (TBSA) between 12/2020-8/2021. Those not surviving the first 24 hours were excluded. Patient demographics and injury characteristics were collected, and resuscitation volumes and timing were recorded prospectively. Patients were categorized by whether their initial 24-hour intake exceeded their Ivy Index, and groups were compared to assess factors associated with over-resuscitation.

**Results:**

During the study period 11 patients met inclusion criteria, and one early mortality was excluded. Patients were predominantly male (70.0%), with mean age 49.9±17.4 years. Burns were primarily due to flame injury, with mean TBSA 41.4±18.6%. Patients required resuscitation volumes of 5.9±1.7 ml/kg/TBSA%, with half surpassing their Ivy Index in the first 24 hours. These patients had larger burns (55.1±17.0% v. 27.7±5.1%) with a significantly higher third degree component (41.4±15.8% v. 15.4±15.2%, p = 0.029). None had diagnosed inhalation injury, and none required abdominal decompression for resuscitation-related compartment syndrome. Observed mortality rate was 30.0%. Patients resuscitating beyond their Ivy Index had significantly higher hourly urine output rates (0.96 v. 0.52 ml/kg, p = 0.024), and hourly urine output was significantly higher among non-survivors as compared to survivors (1.10 v. 0.60 ml/kg, p = 0.033).

**Conclusions:**

Patients with severe burn injury are at high risk for over-resuscitation and associated complications. While traditional teaching instructs a goal of hourly urine output of 0.5-1.0 ml/kg, our study shows that patients resuscitated on the higher end of this range were significantly more likely to surpass their Ivy Index and less likely to survive.

